# 1,3,5-Trichloro-2-methoxy­benzene

**DOI:** 10.1107/S160053680800055X

**Published:** 2008-01-11

**Authors:** Sanjay Telu, Sean Parkin, Larry W. Robertson, Hans-Joachim Lehmler

**Affiliations:** a Department of Occupational and Environmental Health, University of Iowa, 100 Oakdale Campus, 124 IREH, Iowa City, IA 52242-5000, USA; b Department of Chemistry, University of Kentucky, Lexington, KY 40506-0055, USA

## Abstract

The meth­oxy group of the title compound, C_7_H_5_Cl_3_O, is rotated out of the plane of the aromatic ring system, with a dihedral angle of 84.11 (13)°, due to the two bulky *ortho*-chloro substituents.

## Related literature

For similar structures of anisoles with two *ortho*-chloro substituents, see: Rissanen *et al.* (1987 ▶); Weller & Gerstner (1995 ▶); Wieczorek (1980 ▶). For other related literature, see: Brownlee *et al.* (1993 ▶); Curtis *et al.* (1972 ▶); Iimura *et al.* (1984 ▶); Kolehmainen & Knuutinen (1983 ▶); Oswald *et al.* (2005 ▶); Pereira *et al.* (2000 ▶); Rissanen *et al.* (1988 ▶); Vlachos *et al.* (2007 ▶); Zhang *et al.* (2006 ▶).
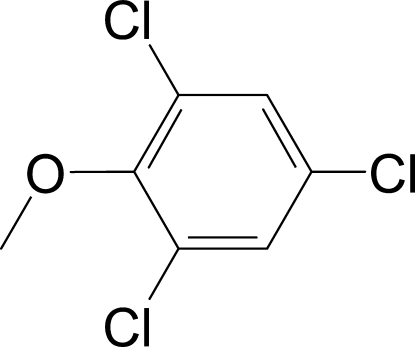


## Experimental

### 

#### Crystal data

C_7_H_5_Cl_3_O
*M* *_r_* = 211.46
Monoclinic, 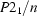

*a* = 14.7538 (5) Å
*b* = 3.9846 (2) Å
*c* = 15.4810 (7) Å
β = 115.5031 (19)°
*V* = 821.42 (6) Å^3^
*Z* = 4
Mo *K*α radiation
μ = 1.05 mm^−1^
*T* = 90.0 (2) K
0.25 × 0.25 × 0.12 mm

#### Data collection

Nonius KappaCCD area-detector diffractometer
Absorption correction: multi-scan (*SCALEPACK*; Otwinowski & Minor, 1997 ▶) *T*_min_ = 0.780, *T*_max_ = 0.885
13489 measured reflections
1888 independent reflections
1558 reflections with *I* > 2σ(*I*)
*R*_int_ = 0.020

#### Refinement

*R*[*F* ^2^ > 2σ(*F* ^2^)] = 0.030
*wR*(*F* ^2^) = 0.070
*S* = 1.13
1888 reflections
101 parameters
H-atom parameters constrained
Δρ_max_ = 0.35 e Å^−3^
Δρ_min_ = −0.31 e Å^−3^

### 

Data collection: *COLLECT* (Nonius, 1998 ▶); cell refinement: *SCALEPACK* (Otwinowski & Minor, 1997 ▶); data reduction: *DENZO-SMN* (Otwinowski & Minor, 1997 ▶); program(s) used to solve structure: *SHELXS97* (Sheldrick, 2008 ▶); program(s) used to refine structure: *SHELXL97* (Sheldrick, 2008 ▶); molecular graphics: *XP* in *SHELXTL* (Sheldrick, 1994 ▶); software used to prepare material for publication: *SHELX97* and local procedures.

## Supplementary Material

Crystal structure: contains datablocks I, global. DOI: 10.1107/S160053680800055X/om2200sup1.cif

Structure factors: contains datablocks I. DOI: 10.1107/S160053680800055X/om2200Isup2.hkl

Additional supplementary materials:  crystallographic information; 3D view; checkCIF report

## Figures and Tables

**Fig. 1.**
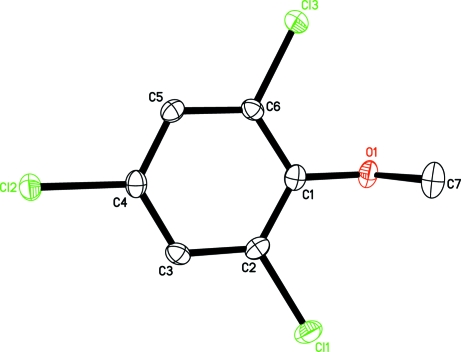
View of the title compound showing the atom-labelling scheme. Displacement ellipsoids are drawn at the 50% probability level.

## supplementary crystallographic information

### Comment

Chlorinated anisoles are persistent environmental pollutants known for their toxicity and tendency to bioaccumulate (Brownlee *et al.*, 1993). Especially anisoles with chlorine substitution in the 2- and 6- position are a well known cause for off-flavors in water, fish and chicken eggs (Brownlee *et al.*, 1993; Curtis *et al.*, 1972; Zhang *et al.*, 2006). The title compound is a major contributor to "cork taint", a well known off-flavor in the wine industry (Pereira *et al.*, 2000; Vlachos *et al.*, 2007). We herin report the molecular structure of the title compound to aid in structure activity relationship (SAR) studies of the toxicity of chlorinated anisoles.

It has been reported that *ortho*-disubstitution causes drastic changes in the spatial arrangement of the methoxy group of chlorinated anisols, both in solution and in the solid state (Kolehmainen & Knuutinen, 1983; Rissanen *et al.*, 1987). In the title compound, the methoxy group was rotated out of the plane of the phenyl ring with a dihedral angle of 84.11 (13)°. The angle was calculated between the plane of the benzene ring (C1 through C6) and the methoxy group (atoms C1,O1,C7). Comparable anisoles have similar dihedral angles ranging from 76.8 to 92.1° (Iimura *et al.*, 1984; Rissanen *et al.*, 1987; Rissanen *et al.*, 1988; Weller & Gerstner, 1995; Wieczorek, 1980). In contrast, the methoxy group of non-*ortho* substituted anisoles lies within the plane of the phenyl ring.

The O—CH_3_ bond length of the title compound (O1—C7: 1.446 (2) Å) was larger compared to non-*ortho* substituted anisoles, which have a mean bond length of 1.422 Å. Furthermore, the C_Ar_—O—CH_3_ bond angle of the title compound (C1—O1—C7: 114.60 (15)°) was smaller compared to non-*ortho* substituted anisoles, which is on average 117.73°. Chlorine disubstitution *ortho* to an aromatic methoxy group had a similar effect on the O—CH_3_ bond length and the C_Ar_—O—CH_3_ bond angles in several structurally related compounds (Iimura *et al.*, 1984; Rissanen *et al.*, 1987; Rissanen *et al.*, 1988; Weller & Gerstner, 1995; Wieczorek, 1980).

Ortho disubstitution forced the methoxy group out of the plane of the aromatic ring, so that the CH_3_ group (C7) of the title compound was located 1.192 (4) Å above and the O1 atom -0.079 (3)Å below the calculated least-squares plane of the benzene ring. In other, structurally related compounds with *ortho* dichloro substitution, the O atoms are positioned 0.030 to 0.133 Å below and the methoxy C atoms 1.119 to 1.252 Å above the calculated last-squares plane (Iimura *et al.*, 1984; Rissanen *et al.*, 1987; Rissanen *et al.*, 1988; Weller & Gerstner, 1995; Wieczorek, 1980). Overall, this difference in the spatial arrangement of the methoxy group is thought to explain the off-flavor of anisoles with *ortho* disubstitution.

### Experimental

Crystals of the title compound suitable for crystal structure analysis were obtained from TCI America (Portland, Oregon, USA.).

### Refinement

H atoms were found in difference Fourier maps and subsequently placed in idealized positions with constrained C—H distances of 0.98 Å (C_Me_H) and 0.95 Å (C_Ar_H) with *U*_iso_(H) values set to either 1.5*U*_eq_ (C_Me_H) or 1.2*U*_eq_ (C_Ar_H) of the attached C atom respectively.

### Crystal data

**Table:**

C_7_H_5_Cl_3_O	*F*_000_ = 424
*M**_r_* = 211.46	*D*_x_ = 1.710 Mg m^−^^3^
Monoclinic, *P*2_1_/*n*	Mo *K*α radiation λ = 0.71073 Å
Hall symbol: -P 2yn	Cell parameters from 2156 reflections
*a* = 14.7538 (5) Å	θ = 1.0–27.5º
*b* = 3.9846 (2) Å	µ = 1.05 mm^−^^1^
*c* = 15.4810 (7) Å	*T* = 90.0 (2) K
β = 115.5031 (19)º	Block, colourless
*V* = 821.42 (6) Å^3^	0.25 × 0.25 × 0.12 mm
*Z* = 4	

### Data collection

**Table:**

Nonius KappaCCD area-detector diffractometer	1888 independent reflections
Radiation source: fine-focus sealed tube	1558 reflections with *I* > 2σ(*I*)
Monochromator: graphite	*R*_int_ = 0.020
Detector resolution: 18 pixels mm^-1^	θ_max_ = 27.5º
*T* = 90.0(2) K	θ_min_ = 1.6º
ω scans at fixed χ = 55°	*h* = −18→19
Absorption correction: multi-scan(SCALEPACK; Otwinowski & Minor, 1997)	*k* = −5→5
*T*_min_ = 0.780, *T*_max_ = 0.885	*l* = −20→19
13489 measured reflections	

### Refinement

**Table:**

Refinement on *F*^2^	Secondary atom site location: difference Fourier map
Least-squares matrix: full	Hydrogen site location: inferred from neighbouring sites
*R*[*F*^2^ > 2σ(*F*^2^)] = 0.030	H-atom parameters constrained
*wR*(*F*^2^) = 0.070	*w* = 1/[σ^2^(*F*_o_^2^) + (0.0244*P*)^2^ + 0.6499*P*] where *P* = (*F*_o_^2^ + 2*F*_c_^2^)/3
*S* = 1.13	(Δ/σ)_max_ = 0.001
1888 reflections	Δρ_max_ = 0.35 e Å^−^^3^
101 parameters	Δρ_min_ = −0.31 e Å^−^^3^
Primary atom site location: structure-invariant direct methods	Extinction correction: none

### Special details

**Table:**

Geometry. All e.s.d.'s (except the e.s.d. in the dihedral angle between two l.s. planes) are estimated using the full covariance matrix. The cell e.s.d.'s are taken into account individually in the estimation of e.s.d.'s in distances, angles and torsion angles; correlations between e.s.d.'s in cell parameters are only used when they are defined by crystal symmetry. An approximate (isotropic) treatment of cell e.s.d.'s is used for estimating e.s.d.'s involving l.s. planes.
Refinement. Refinement of *F*^2^ against ALL reflections. The weighted *R*-factor *wR* and goodness of fit *S* are based on *F*^2^, conventional *R*-factors *R* are based on *F*, with *F* set to zero for negative *F*^2^. The threshold expression of *F*^2^ > 2σ(*F*^2^) is used only for calculating *R*-factors(gt) *etc*. and is not relevant to the choice of reflections for refinement. *R*-factors based on *F*^2^ are statistically about twice as large as those based on *F*, and *R*- factors based on ALL data will be even larger.

### Fractional atomic coordinates and isotropic or equivalent isotropic displacement parameters (Å^2^)

**Table:**

	*x*	*y*	*z*	*U*_iso_*/*U*_eq_	
Cl1	0.65436 (3)	0.31728 (13)	0.83292 (3)	0.02202 (13)	
Cl2	0.36963 (3)	0.90882 (13)	0.91077 (3)	0.01965 (12)	
Cl3	0.32350 (3)	0.84337 (13)	0.54775 (3)	0.02053 (13)	
O1	0.51708 (9)	0.4751 (4)	0.63059 (9)	0.0189 (3)	
C1	0.48575 (13)	0.5887 (5)	0.69659 (13)	0.0161 (4)	
C2	0.54027 (13)	0.5242 (5)	0.79404 (13)	0.0161 (4)	
C3	0.50643 (13)	0.6209 (5)	0.86111 (13)	0.0162 (4)	
H3	0.5447	0.5753	0.9273	0.019*	
C4	0.41531 (14)	0.7858 (5)	0.82874 (13)	0.0164 (4)	
C5	0.35818 (13)	0.8564 (5)	0.73309 (13)	0.0158 (4)	
H5	0.2958	0.9705	0.7126	0.019*	
C6	0.39426 (13)	0.7561 (5)	0.66748 (12)	0.0147 (4)	
C7	0.58710 (16)	0.6940 (6)	0.61549 (15)	0.0253 (5)	
H7A	0.5565	0.9156	0.5951	0.038*	
H7B	0.6039	0.5988	0.5659	0.038*	
H7C	0.6483	0.7159	0.6753	0.038*	

### Atomic displacement parameters (Å^2^)

**Table:**

	*U*^11^	*U*^22^	*U*^33^	*U*^12^	*U*^13^	*U*^23^
Cl1	0.0146 (2)	0.0252 (3)	0.0231 (2)	0.00407 (19)	0.00509 (19)	−0.0021 (2)
Cl2	0.0178 (2)	0.0266 (3)	0.0163 (2)	0.00018 (18)	0.00906 (18)	−0.00204 (18)
Cl3	0.0190 (2)	0.0279 (3)	0.0139 (2)	0.00294 (19)	0.00632 (18)	0.00251 (18)
O1	0.0171 (7)	0.0230 (8)	0.0205 (7)	−0.0022 (6)	0.0119 (6)	−0.0046 (6)
C1	0.0160 (9)	0.0152 (10)	0.0188 (9)	−0.0037 (7)	0.0092 (7)	−0.0036 (7)
C2	0.0123 (8)	0.0142 (10)	0.0208 (9)	−0.0003 (7)	0.0061 (7)	−0.0008 (7)
C3	0.0152 (9)	0.0177 (10)	0.0129 (8)	−0.0030 (7)	0.0035 (7)	−0.0009 (7)
C4	0.0177 (9)	0.0160 (10)	0.0184 (9)	−0.0037 (7)	0.0106 (8)	−0.0031 (7)
C5	0.0128 (8)	0.0155 (10)	0.0192 (9)	−0.0002 (7)	0.0069 (7)	0.0005 (7)
C6	0.0138 (9)	0.0168 (10)	0.0120 (8)	−0.0023 (7)	0.0040 (7)	0.0002 (7)
C7	0.0273 (11)	0.0255 (11)	0.0310 (11)	−0.0054 (9)	0.0201 (9)	−0.0052 (9)

### Geometric parameters (Å, °)

**Table:**

C1—O1	1.367 (2)	Cl3—C6	1.7264 (18)
C1—C2	1.394 (3)	C4—C5	1.381 (3)
C1—C6	1.395 (3)	C5—C6	1.393 (3)
C2—C3	1.386 (3)	C5—H5	0.9500
C2—Cl1	1.7329 (19)	O1—C7	1.446 (2)
C3—C4	1.382 (3)	C7—H7A	0.9800
C3—H3	0.9500	C7—H7B	0.9800
Cl2—C4	1.7447 (18)	C7—H7C	0.9800
			
O1—C1—C2	121.63 (16)	C4—C5—H5	120.8
O1—C1—C6	120.55 (16)	C6—C5—H5	120.8
C2—C1—C6	117.70 (16)	C5—C6—C1	121.46 (16)
C3—C2—C1	122.21 (17)	C5—C6—Cl3	118.74 (14)
C3—C2—Cl1	118.77 (14)	C1—C6—Cl3	119.79 (14)
C1—C2—Cl1	119.02 (14)	C1—O1—C7	114.60 (15)
C4—C3—C2	117.92 (17)	O1—C7—H7A	109.5
C4—C3—H3	121.0	O1—C7—H7B	109.5
C2—C3—H3	121.0	H7A—C7—H7B	109.5
C5—C4—C3	122.34 (17)	O1—C7—H7C	109.5
C5—C4—Cl2	118.33 (14)	H7A—C7—H7C	109.5
C3—C4—Cl2	119.33 (14)	H7B—C7—H7C	109.5
C4—C5—C6	118.36 (17)		
			
O1—C1—C2—C3	−176.17 (17)	Cl2—C4—C5—C6	−179.55 (14)
C6—C1—C2—C3	−0.1 (3)	C4—C5—C6—C1	−0.1 (3)
O1—C1—C2—Cl1	4.0 (3)	C4—C5—C6—Cl3	−179.94 (14)
C6—C1—C2—Cl1	−179.91 (14)	O1—C1—C6—C5	176.18 (17)
C1—C2—C3—C4	0.1 (3)	C2—C1—C6—C5	0.1 (3)
Cl1—C2—C3—C4	179.96 (15)	O1—C1—C6—Cl3	−4.0 (3)
C2—C3—C4—C5	−0.2 (3)	C2—C1—C6—Cl3	179.92 (14)
C2—C3—C4—Cl2	179.51 (15)	C2—C1—O1—C7	−86.2 (2)
C3—C4—C5—C6	0.1 (3)	C6—C1—O1—C7	97.9 (2)
